# Kinetic Analysis of the Reaction of Silver with Elemental Sulfur in Mineral Insulating Oil

**DOI:** 10.3390/ma18163771

**Published:** 2025-08-12

**Authors:** Dejan Kolarski, Jelena Lukić, Jelena Janković, Sandra Glišić

**Affiliations:** 1 Nikola Tesla Electrical Engineering Institute, 11040 Belgrade, Serbia; lukicjelena@ieent.org (J.L.); jelena.jankovic@ieent.org (J.J.); 2 Faculty of Technology and Metallurgy, University of Belgrade, 11000 Belgrade, Serbia; sglisic@tmf.bg.ac.rs

**Keywords:** elemental sulfur, kinetics model, diffusion, mineral insulating oil, silver corrosion

## Abstract

Elemental sulfur (S_8_) reacts readily with silver, forming highly conductive silver sulfide on silver-coated components of on-load tap changers (OLTCs), forming a highly conductive silver sulfide film at the surface of an OLTC, which can lead to the failure of critical components in power transformers. This study investigates the reaction between metallic silver and elemental sulfur dissolved in mineral insulating oil across temperatures from 60 °C to 180 °C. The process involves three stages: the diffusion of sulfur through oil, surface reaction, and product diffusion. For low-viscosity oil, diffusion is not the limiting factor, and sulfur does not react immediately on the silver’s surface, suggesting possible adsorption or intermediate formation. A kinetic analysis revealed that the reaction follows first-order kinetics, with a change in mechanism above 150 °C. The reaction follows the Arrhenius equation in two separate regions: 60–150 °C and 150–180 °C. Activation energy was calculated as 23.67 kJ mol^−1^, and it can be concluded that the reaction is controlled by the diffusion of sulfur through mineral oil, and at higher temperatures (150 °C to 180 °C), the calculated activation energy is 160.69 kJ mol^−1^, which leads to the conclusion that the combined chemisorption and diffusion through a silver sulfide–oil interface becomes the new limiting factor.

## 1. Introduction

The problem of corrosive sulfur is well known in the power industry, and it remains a worldwide concern, since the presence of corrosive sulfur in power transformers is often identified as a reason for sudden and unforeseen failures of power transformers. Some sulfur compounds, such as elemental sulfur (S_8_), mercaptans, and disulfides such as dibenzyl disulfide (DBDS), which may be present in mineral transformer oil, can interact with the silver-coated components of on load tap changers (OLTCs), creating a semi-conductive film of silver sulfides on their surfaces [1,2,3,4]. Depending on the type of the present sulfur compound, operational temperature, oxygen content, and electrical fields, some sulfur compounds have a higher affinity to react with copper, while other sulfur compounds have a higher affinity to react with the silver-plated components in power transformers [5,6,7,8,9,10]. Meanwhile, DBDS has been considered as a dominant corrosive sulfur compound in mineral oils causing copper sulfide deposition in paper-wrapped copper conductors, which was thoroughly investigated in the last decade, and mechanisms of the DBDS reaction with copper have been proposed [7,8,9,10,11,12,13]. After copper corrosion was induced by DBDS, elemental sulfur appeared as the main cause of corrosion towards silver surfaces in a transformer [1]. The formation of elemental sulfur, which is not normally present in mineral insulating oils, occurred as a result of the oil regeneration processes of used mineral insulating oils with reactivating adsorbents during the reclamation process when reactivating procedures were applied, using high-temperature combustion process up to 500 °C or higher. Elemental sulfur is created by the cracking of various sulfur-containing species retained in the reactivated adsorbent in the later stages of oil regeneration of contaminated treated oil in the power transformer [1,2,4,14,15,16]. Due to the high affinity of S_8_ to react with silver and the large amount of oil in the transformer compared to the small surface of the OLTC’s silver-plated contacts, only a few mg/kg of S_8_ in the oil is sufficient to create silver corrosion at a low operating temperature [15,17,18,19,20]. The reaction between metallic silver and sulfur has been investigated intensively in the past years due to a process known as silver tarnishing [21,22,23]. Since the silver tarnishing process occurs at low temperatures, the main focus has been investigating this reaction at low temperatures, and a consistent finding is that the diffusion is the rate-determining step in this process [19,21,22]. In this study, the reaction between metallic silver and elemental sulfur dissolved in mineral insulating oil has been investigated across a broad temperature range from 60 °C up to 180 °C, that incorporates normal operating temperatures of power transformers, higher operating temperatures due to overloading conditions, and overheating that may occur during operation.

## 2. Materials and Methods

### 2.1. Materials and Analysis

New mineral insulating oil Nynas Nytro 4000X (Naphthenics AB, Stockholm, Sweden) was used; it is non-corrosive, and it was spiked with a 5.4 ppm of S_8_ using reagent-grade sulfur powder, which was procured from Sigma Aldrich (St. Louis, MO, USA). Silver strips also purchased from Sigma Aldrich were prepared according to the ASTM D1275-15 standard [24]. To clean the silver strips, their surfaces were polished using a polishing paste as a fine abrasive, after which their surfaces were washed using laboratory-grade acetone and then placed in a hexane bath to prevent any atmospheric reactions.

The concentrations of S_8_ at specified sampling times were determined using an Agilent 7890B gas chromatograph, Agilent Technologies, Inc., Wilmington, DE, USA, equipped with an electron capture detector (limit of detection, LOD = 0.05 mg/kg; limit of quantification, LOQ = 0.18 mg/kg). Oil samples were prepared using analytical-grade toluene, which was also procured from Sigma Aldrich, following the IEC TR 62697-3/2018 standard [25]. The calibration of the instrument with known concentrations of S_8_ in oil has been prepared, ranging from 0.5 ppm to 5.5 ppm of S_8_, with a correlation factor of 0.99990.

Determination of the morphological and chemical composition of silver plates before and after the experiments at selected temperatures was performed by a scanning electron microscope (SEM), a model JEOL JSM-6610LV, JEOUL Ltd., Tokyo, Japan, equipped by an EDS, and an Oxford X-Max energy dispersive spectrometer. The analyses were performed under high vacuum, with an acceleration voltage of 20 kV. The detection limit for elements was ∼0.1 wt.%.

### 2.2. Experimental Set Up

Aging experiments were carried out according to the ASTM D1275-15 standard at eight different temperatures: 60 °C, 80 °C, 100 °C, 120 °C, 140 °C, 150 °C, 165 °C, and 180 °C, with durations adjusted based on the temperature and expected reaction rate to achieve a conversion rate of 80% and higher. At higher temperatures, samples were taken at intervals of 15 to 30 min over one to two days, while at lower temperatures, samples were taken at intervals ranging from several hours to 24 h for up to twenty-one days of aging; to prevent any atmospheric influence during the experiment, the headspace was purged with argon, and the bottles were sealed. The initial concentration of sulfur in all experiments was 5.4 ppm.

## 3. Results and Discussion

High reactivity of elemental sulfur (S_8_) at low temperatures was observed in laboratory tests and supported by service experiences. It was observed that the deposition of silver sulfide can start from low temperatures (room and slightly higher, 40 °C if S_8_ is present in high concentrations as of 10–20 ppm). It was confirmed in all cases that oils did not contain a metal passivator, thus excluding possible interference in the corrosion test by its presence, although previous publications and service experiences have shown that a metal passivator is not efficient to protect silver surfaces against sulfur corrosion.

### 3.1. Analysis of Adsorption Process and Rate Determining Step

The aim of this investigation is to analyze the tarnishing process between silver plates and S_8_ in mineral transformer oil and to evaluate each step both theoretically and experimentally. The goal was to establish the slowest step, rate-determining, in this overall heterogeneous reaction, which could compromise several consecutive and/or simultaneous steps. For this purpose, the proposed mechanism and scenario by Foley and coworkers [21] will be applied on experimental data derived from this study.

- First step—diffusion in solution: The S_8_ in mineral oil must diffuse to the surface of the silver plate in order to react with silver. A theoretical and experimental study of the free convection derived by Wagner’s Equation (1) is used for the calculation of the rate of solution per unit area, *n*/*A*, depending on the diffusion coefficient, *D*, Equation (2), and the Sutherland–Einstein relation [21]. The concentration in the solution is given by *c*, ν is the kinematic viscosity of the medium of density *ρ*, and *H* is the vertical height of the plate. *∆ρ* is the density difference between the solution with concentration c and the solvent itself:
  (1) $$\frac{n}{A}=0.726*D*c{\left(\frac{g}{\nu *D*H}*\frac{\Delta \rho }{\rho }\right)}^{\frac{1}{4}}$$
  (2) $$D=k*T*\frac{1}{6*\pi *\eta *r}$$
  where *k* = R/N = 1.380 × 10^−16^ ergs deg.^−1^, R is the universal gas constant, N is Avogadro’s number, *T* is temperature in °C, g is the gravitational acceleration 9.81 m s^−2^, η is viscosity in poise, and *r* = 2.70 × 10^−8^ cm. The “radius” of the sulfur molecule, *r*, was calculated based on an eight-membered ring with an S—S bond distance of 2.08A [21].
- Second step—adsorption on silver plate: Adsorption is usually used in the literature to describe the overall process by which the S_8_ makes the transition from the solution to the silver surface, or the S_8_ reaction with silver molecules giving silver sulfide as reaction product. For this reason, the second step in adsorption on a silver plate could be adsorption on a surface or it might be the formation of a reaction intermediate. Also, the adsorption of other compounds, which could be found in the transformer oil through oxidation or aging of the oil, must be considered and analyzed in a kinetic model.
- Third step—diffusion in the reaction product: The diffusion of metal outward and of sulfur inward through the reaction product film has been considered by many workers as rate-determining for reactions of this type. Wagner’s hypothesis is based on the diffusion of cations and electrons rather than of neutral atoms through a reaction product film, and a good agreement between calculated and experimental reaction rates has been obtained in several cases.

**Figure 1:**
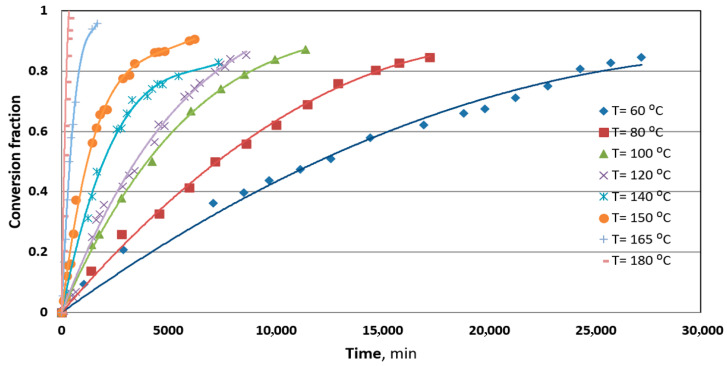
The conversion fraction of the reaction between metallic silver and elemental sulfur S_8_ in mineral oil at different temperatures.

The purpose of this work, then, was to evaluate each of these steps, if possible, quantitatively, so that the mechanism of the reaction between a silver plate and S_8_ in mineral transformer oil may be represented with accuracy. Figure 1 shows the experimental results of the conversion fraction of sulfur during the time of reaction. The curves are fitting experimental data with the polynomial of a second order, and it could be realized that at temperatures above 150 °C, the reaction of a silver plate and elemental sulfur is very fast, suggesting that the diffusion trough dissolution of mineral oil could not be a limiting step.

Table 1 shows the values of density, viscosity, and diffusivity at various temperatures for the used mineral transformer oils.

**Figure 2:**
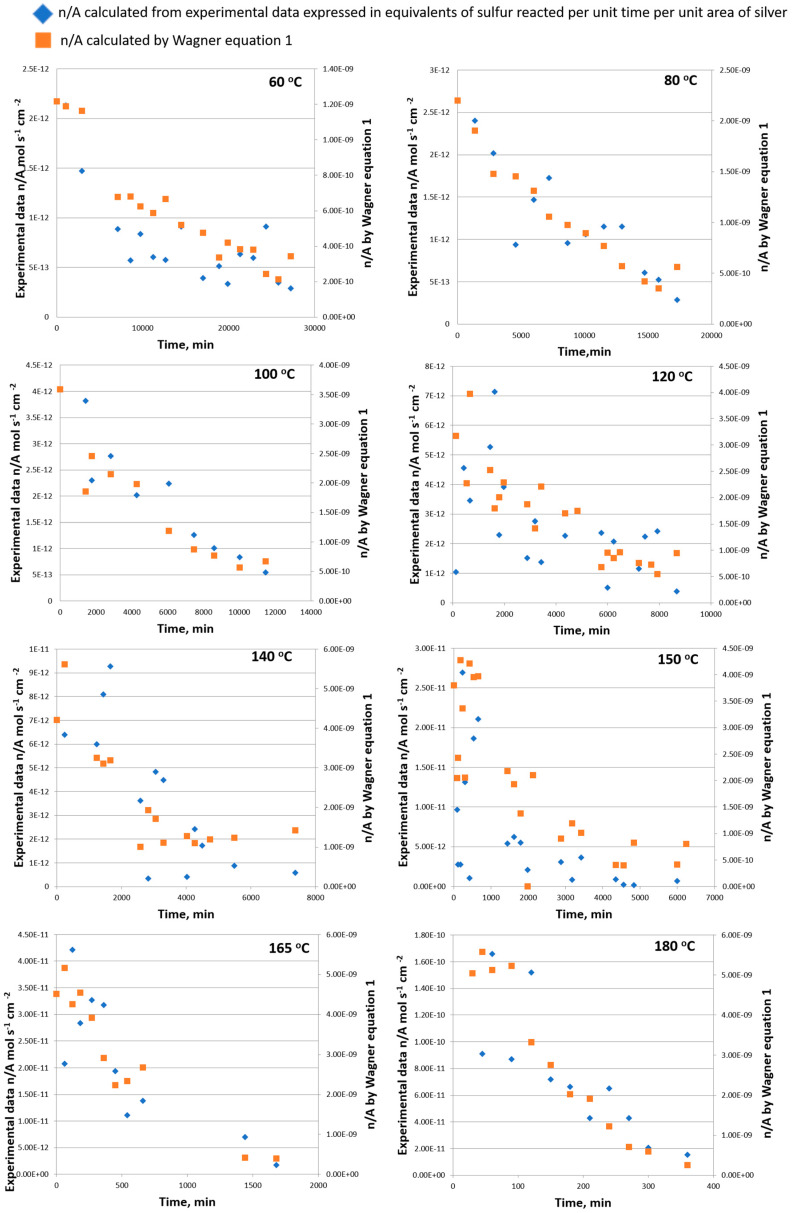
The observed sulfiding rate of silver compared with the calculated diffusion rate of sulfur in the temperature range of 60–180 °C.

The data obtained in this study, calculated in equivalents of sulfur reacted per unit time per unit area of silver, written as n/A, are plotted against the time in Figure 2 at different temperatures and shown with blue markers. The decrease in the rate with an increase in time is at once conspicuous. If the rate n/A is governed by diffusion supported by convection, it should be predictable by Equation (1) and entirely independent data, as shown with orange markers in Figure 2. From the obtained data in Figure 2, the values of n/A obtained by Wagner Equation (1) are around 100 times higher than the n/A obtained as a reaction rate derived from experimental data for all analyzed temperatures. This suggests that diffusion governed by convection is rate-determining in the analyzed system at analyzed temperatures. Also, by increasing the temperature difference in n/A calculated by diffusion, values obtained as a reaction rate become less diverse (difference is becoming 10 times larger), confirming also that diffusion is a controlling step in this process (Figure 2). The increase in the rate of reaction with temperature can be explained very well by the increase in diffusion supported by convection. Foley and co-authors [21] concluded that diffusion is not the rate-determining influence in solvents of low viscosity, such as the oil used in this study, and one of the other steps in the overall reaction mechanism has assumed this role.

Foley and co-authors [21] also found that when rates calculated by the Wagner equation reached limits of about 6−8 × 10^−10^ equiv. cm^−2^ s^−1^, it appears that the diffusion ceased to be the rate-determining influence. Analyzing the data in our study with low viscosity, low-density mineral oil observed in the temperature range 60–180 °C, where the rates calculated by the Wagner equation gave values in the range of 5 × 10^−9^–3 × 10^−10^ equiv. cm^−2^ s^−1^, could be concluded by the Foley observation that the viscosity change was not related to the sulfiding rate.

The second step, the adsorption step, is usually a fast step and occurs in the reaction series. But when favorably adsorbed compounds are present on the silver plate surface, it could be the slow and rate-determining step [21]. The silver surface can be poisoned by the adsorption of sulfides and disulfides, which are molecules whose structure resembles sulfur from the standpoint of coordination electrons [21]. Furthermore, polar groups could be adsorbed on the silver surface, preventing the reaction to some degree and making the adsorption of sulfur the slow step in the whole process. Thus, this competitive mechanism is analyzed by Foley and co-authors, and they found that if there are some other molecules which could competitively adsorb on the silver surface, the calculated n/A values, their average rates, should be higher than 0.61 × 10^−10^ equiv. cm^−2^ s^−1^. A comparison of rates in this paper illustrates that there are probably other molecules which could interrupt the sulfur adsorption on silver’s surface and slow down this step.

The third possible step is the diffusion of the reactants through the silver sulfide reaction product. Wagner, Reinhold, and Móhring studied the reaction between silver and sulfur in the temperature range of 130 °C to 170 °C and calculated at 60 °C and concluded, that, with a solution as viscous as the mineral oil used in this study, the film diffusion could not be the rate-determining step [26,27]. Usually this is a rapid step, and this step could be rate-determining only if the viscosity is decreased significantly.

### 3.2. SEM and EDS Analysis as Confirmation of Silver Sulfide Formation on Silver Plate

**Figure 3:**
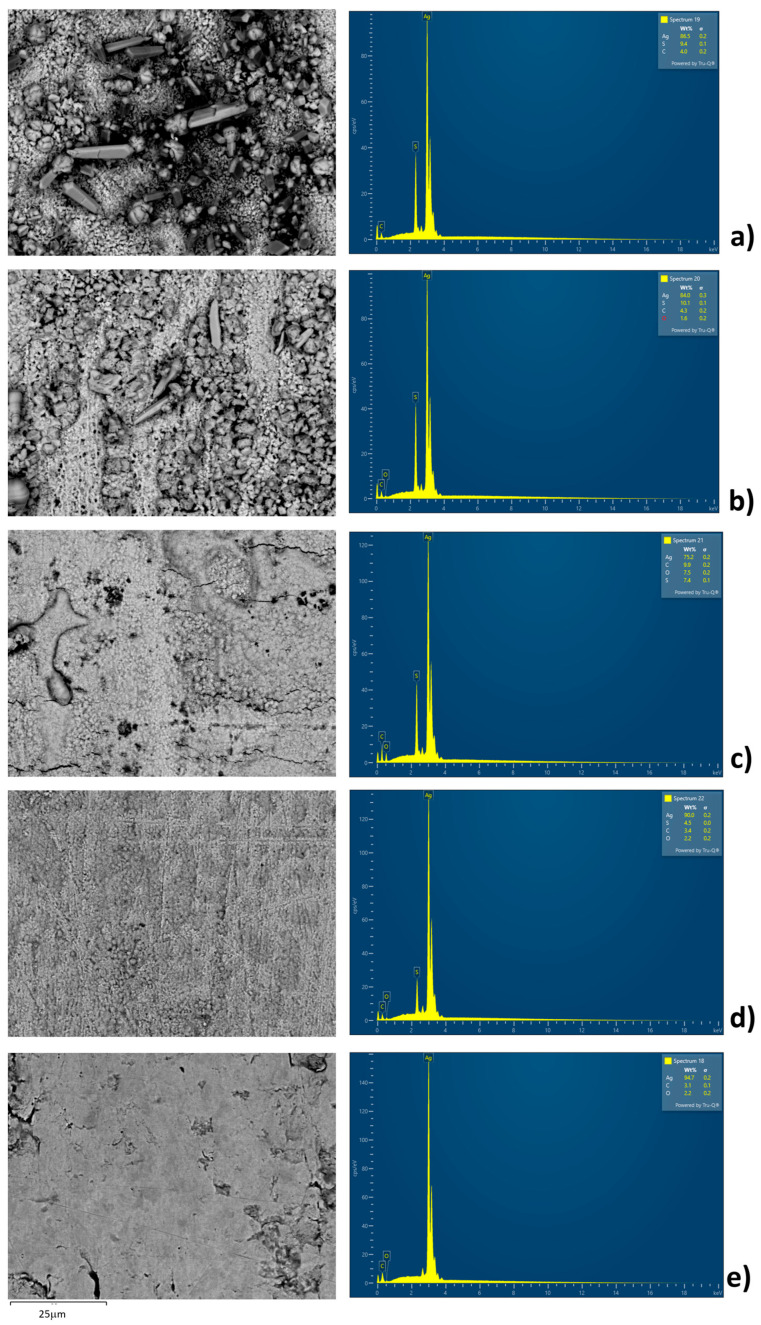
SEM images and EDS spectra of silver plate surface after the experiments performed at (**a**) 80 °C, (**b**) 100 °C, (**c**) 150 °C, (**d**) 180 °C, and (**e**) new unused silver plate.

**Figure 4:**
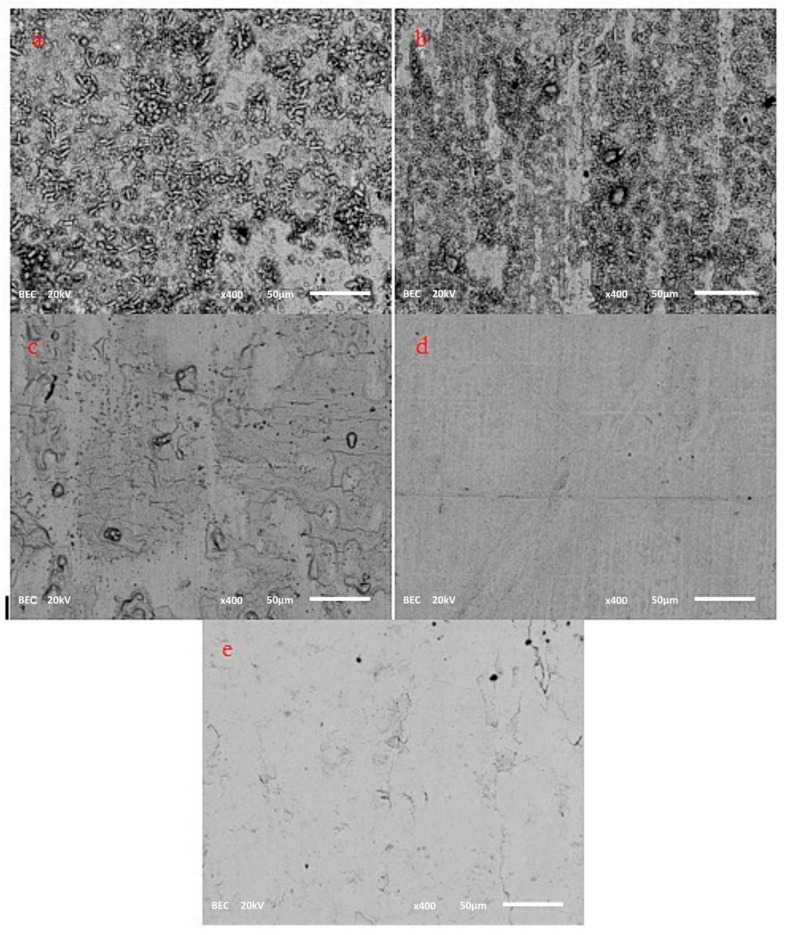
SEM images of silver plate surface after the experiments performed at (**a**) 80 °C, (**b**) 100 °C, (**c**) 150 °C, (**d**) 180 °C, and (**e**) new unused silver plate.

EDS analysis and SEM analysis are performed in order to confirm the presence of sulfur at the surface of the silver plate (Table 2 and Figure 3 and Figure 4). At lower temperatures, crystals at the surface of silver plates are clearly visible. At 80 °C, EDS analysis confirmed the presence of Ag, C, and S, while oxygen was not detected. As the temperature increases to 100 °C, crystals remain observable on the Ag plate surface but in significantly smaller quantities compared to 80 °C, and EDS analysis confirms the presence of Ag, C, S, and O. With further temperature increase to 150 °C, crystals are no longer visible (Figure 3). The surface appears melted but inhomogeneous, showing pronounced topographical variations (Figure 4). EDS analysis confirms the presence of Ag, C, S, and O; a significant rise in the percentages was observed for C, S, and O, while a decrease was noted in the Ag content. It should be noted that the quantification of oxygen and carbon via EDS analysis carries a lower degree of accuracy compared to lower temperatures. At 180 °C, the Ag plate’s surface resembles that of the surface at 150 °C, but it is more uniform and without major topographical variations. EDS analysis confirms the presence of Ag, C, S, and O, although their concentrations, particularly those of C, S, and O, have notably decreased compared to the experiment at 150 °C. EDS analysis of a new, unused plate confirms the presence of Ag, C, and O. It is possible that at higher temperatures the silver sulfide deposits detached from the silver plate and were mitigated in the mineral oil. This phenomenon was observed in practice when multilayer-flakes Ag_2_S deposits were formed, which have a tendency to flake off the silver surface [15,18,28,29,30]. This is the worst scenario in terms of the risk of power transformer failure. This analysis helps to understand the process of diffusion of the elemental sulfur to the surface of the silver plate, the free places on the silver plate for further sulfur adsorption, and the diffusion of the reaction product from the silver plate. The obtained data and EDS analysis confirm that at higher temperatures, above 150 °C, the amount of elemental sulfur adsorbed on the silver plate is a much faster process because the product detaching from the plate surface and from the film formed.

### 3.3. Kinetics Analysis

In the following section, kinetics models are described to evaluate the kinetics of a reaction between metallic silver and elemental sulfur dissolved in mineral insulating oil. The experimental data obtained from experimental measurements were analyzed using first-order and second-order kinetic models in a batch reactor, after which the temperature dependence of the reaction was investigated using the Arrhenius plot.

The rate of the reaction between sulfur and metallic silver increased with an increase in the temperature (Figure 1). Given that there was no stirring of the oil during the experiments, it can be assumed that the diffusion of sulfur through the oil layer does not play a significant role in the overall reaction rate, which was confirmed by previous analysis. The increase in the rate of reaction with temperature in a system without stirring can be explained by the increase in diffusion supported by free convection in relation to the viscosity and density decrease. When sulfur molecules diffuse through the oil towards the silver plate, a concentration boundary layer forms near the surface of the plate. Within this layer, the concentration of sulfur molecules changes from the bulk concentration in the oil to the concentration near the surface of the silver metal plate where the reaction occurs.

Since sulfur is dissolved in mineral insulating oil, the oil’s viscosity can further restrict the mobility of sulfur molecules at lower temperatures, and the oil becomes more viscous, enhancing the diffusional barrier. As the temperature increased, the kinetic energy of molecules increased, and sulfur molecules became more mobile, reaching the silver surface more quickly and resulting in a faster reaction. This led, together with a further viscosity decrease and reduced diffusion barrier, to a faster conversion rate [31,32].

**Figure 5:**
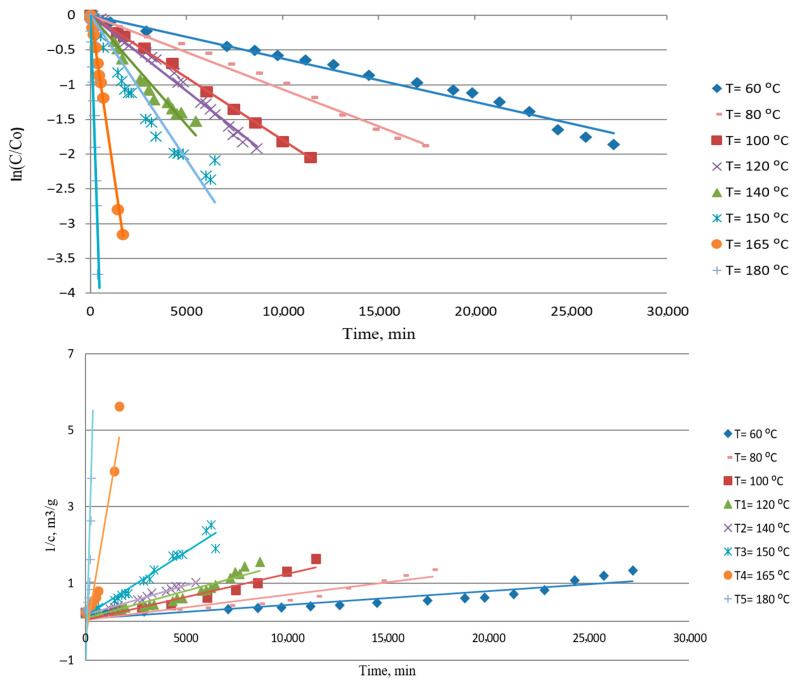
First-order (**top**) and second-order (**bottom**) linear kinetics model for reaction between metallic silver and sulfur in mineral insulating oil.

The first-order kinetic model and the second-order kinetic model for the batch process are presented by Equations (3) and (4), and agreement with model and experimental data is shown in Figure 5:

(3) $$-ln\frac{C}{C_{0}}=k*t$$

(4) $$\frac{1}{C}=\frac{1}{C_{0}}+k*t$$

where *C* is the sulfur concentration (g m^−3^), *C*_0_ is the initial sulfur concentration (g m^−3^), *k* is the first-order constant (min^−1^) or the second-order constant (m^3^ g^−1^ min^−1^), Equation (4), and *t* is the time (min).

The kinetic parameters shown in Table 3 were obtained through the first-order plot and second-order plot. It could be seen from Table 3 that a better regression coefficient (0.9249–0.9984) is obtained for a first-order kinetic model than for a second-order kinetic model (0.6097–0.9840), and a first-order fit better experimental data for all analyzed experiments, which is expected given the large surface area of the silver plate and the low concentration of sulfur dissolved in the oil.

Based on the obtained values of the reaction rate constants at different temperatures, the activation energies for the observed reaction between sulfur dissolved in mineral insulating oil and metallic silver can be calculated using the Arrhenius equation:

(5) $$k=A*e^{-\frac{Ea}{RT}}$$

where *A* is a pre-exponential factor (same units as k), *Ea* (J mol^−1^) is an activation energy for the reaction, *R* is the universal gas constant (equal to 8.314 J K^−1^ mol^−1^), and *T* is the temperature in *K*.

**Figure 6:**
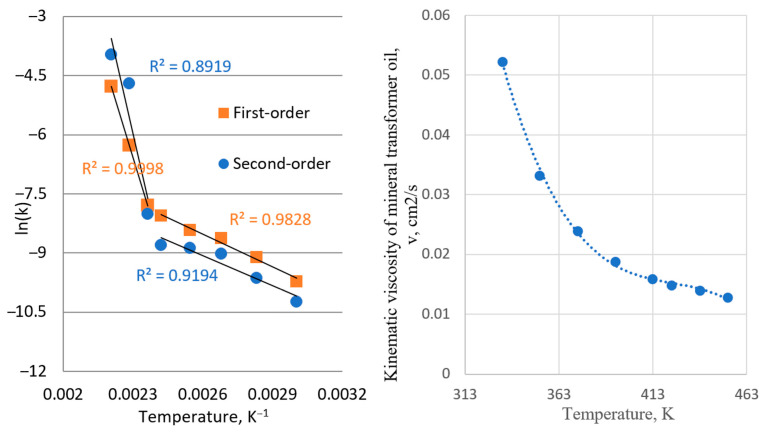
Arrhenius plot from kinetic parameters obtained from first-order kinetic (**left**) and dependence of the kinematic viscosity of mineral transformer oil on temperature (**right**). For the purpose of the kinetic parameters presented by the Arrhenius equation, the temperature on the figure will be presented in Kelvin degree, K.

In Figure 6, it can be seen that the reaction follows the Arrhenius equation in two separate temperature regions: first, at a lower temperature ranging from 60 °C to 150 °C, and second, at a higher temperature region ranging from 150 °C to 180 °C. Calculated values of the pre-exponential factor and activation energy for both periods are given in Table 4.

The obtained transition can be explained by the values of kinematic viscosity, which have the same sharp change in values after temperatures of 140 °C. According to the literature findings, values of activation energy for diffusion-controlled reactions are typically lower compared to those which are controlled by some other steps [31,33,34,35,36]. The obtained values for activation energy for both first-order and second-order reaction rates are similar, 23.67 kJ mol^−1^ and 21.10 kJ mol^−1^, at a lower temperature range, while obtained pre-exponential factors were considerably different. This confirms that the reaction is diffusion-controlled in the lower temperature region and suggests that the reaction is catalyzed by the silver surfaces, possibly implying a combined diffusion through oil–silver sulfide interface and chemisorption.

Chemisorption on metal and film surfaces significantly influences the pre-exponential factor (*A*) and activation energy (*Ea*) in the Arrhenius equation. Chemisorption involves strong chemical bonding between the adsorbate and the surface, leading to higher reaction probabilities compared to physisorption. The value of *A* depends on surface coverage, adsorption site density, and the reaction pathway [35]. For transition metals, *A* can range from 10^9^ to 10^13^ s^−1^, depending on the reaction mechanism. Chemisorption typically has a higher *Ea* than physisorption due to the energy required to break and form chemical bonds. For metal surfaces, *Ea* varies widely, and for sulfur adsorption on Ag: ~150–250 kJ mol^−1^.

**Figure 7:**
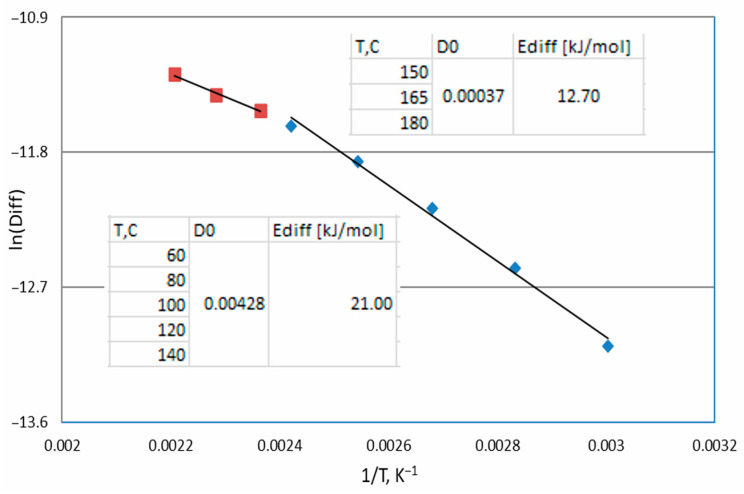
Arrhenius plot of diffusion values.

Thin films can alter *Ea* due to electronic effects, surface defects, and diffusion barriers. In thin films and diffusion-controlled surface reactions, diffusion plays a crucial role in reaction kinetics. If the diffusion coefficient (calculated by Equation (2)), analyzed as the activation energy from the Arrhenius equation (*E*diff, shown in Figure 7), is lower than *Ea* (Table 4), this means that mass transport can be the rate-limiting step. Film thickness and substrate composition affect the adsorption strength and reaction rates.

## 4. Conclusions

Elemental sulfur (S_8_) is highly reactive towards silver and can react with silver-coated components of on-load tap changers, forming a highly conductive silver sulfide film at the surface of the OLTC, which can lead to the failure of critical components in power transformers. In this study, the reaction between metallic silver and elemental sulfur dissolved in mineral insulating oil was investigated at different temperatures, ranging from 60 °C to 180 °C. The three step adsorption phenomena is analyzed: diffusion of mineral oil to the plate surface, reaction on the surface, and diffusion of the products in the mineral oil. Based on the obtained results, it can be concluded that for the investigated mineral oils that have a low viscosity, diffusion in oil is not a rate-limiting step. Otherwise, sulfur does not react immediately upon its arrival at the silver surface or at the silver sulfide–oil interface. This might be a competitive adsorption phenomenon, or it might be the formation of a reaction intermediate. The EDS analysis and SEM analysis shows the formation of crystals, probably of silver sulfide, at the surface of the silver plate at temperatures up to 150 °C, while at higher temperatures above 150 °C, crystals detached from the silver plate, most probably migrating to the mineral oil in the form of particles. Therefore, the whole process could be analyzed as one and a kinetic analysis of the first and second order was applied. It was found that the reaction follows a first-order kinetics model, with a change in the mechanism at temperatures above 150 °C. The observed reaction follows the Arrhenius equation in two separate temperature regions: first, at a lower temperature region ranging from 60 °C to 150 °C, and second, at a higher temperature region ranging from 150 °C to 180 °C. Using the Arrhenius plot, the activation energy of the reaction was determined: at lower temperatures (below 150 °C), the activation energy is 23.67 kJ mol^−1^ for the first order, and at higher temperatures (150 °C to 180 °C), the calculated activation energy is 160.69 kJ mol^−1^. At lower temperatures, ranging from 60 °C to 150 °C, the process is controlled by the diffusion of sulfur through mineral oil to the silver surface. However, at higher temperatures above 150 °C, the reaction exhibits a significant increase in the pre-exponential factor (*A*), indicating a change in mechanism; the limiting factor is no longer diffusion through the oil but through the silver sulfide film formed on the silver surface. The strength of the chemisorption of sulfur on silver increases the activation energy of the reaction. This transition suggests a change from a diffusion-controlled reaction to a reaction primarily governed by the interplay of diffusion through the silver sulfide film and the chemisorptive interactions between silver and sulfur.

## Figures and Tables

**Table 1:** Data of density and viscosity at different temperatures for the used transformer mineral oil.

T, °C	Kinematic Viscosity, v, cm^2^ s^−1^	Density, kg m^−3^	Dynamic Viscosity, pas s	Diffusivity, cm^2^ s^−1^
60	0.052272	840	4.39 × 10^–2^	2.06 × 10^−6^
80	0.033233	830	2.76 × 10^−2^	3.47 × 10^−6^
100	0.023919	820	1.96 × 10^−2^	5.16 × 10^−6^
120	0.018837	805	1.52 × 10^−2^	7.03 × 10^−6^
140	0.015838	790	1.25 × 10^−2^	8.95 × 10^−6^
150	0.014797	782	1.16 × 10^−2^	9.92 × 10^−6^
165	0.013964	775	1.08 × 10^−2^	1.10 × 10^−5^
180	0.012744	765	9.75 × 10^−3^	1.26 × 10^−5^

**Table 2:** Elemental content of surface on the silver plate after experiments at four temperatures and on unused silver plate.

Silver Plate After Experiment Performed at Temperature T, °C	Content of Elements, wt.%			
	Ag	C	O	S
New silver plate	94.7	3.1	2.2	n.d. ^1^
80	86.5	4.0	n.d. ^1^	9.4
100	84.0	4.3	1.6	10.1
150	75.2	9.9	7.5	7.4
180	90.0	3.4	2.2	4.5

1 n.d.—not detected.

**Table 3:** Kinetic parameters for reaction between metallic silver and sulfur in mineral insulating oil.

Temperature, °C	Parameters			
	First-Order Reaction Rate Constant, (min^−1^)	R^2^	Second-Order Reaction Rate Constant, (m^3^ g^−1^ min^−1^)	R^2^
60	0.000060	0.9762	0.000036	0.8467
80	0.000110	0.9886	0.000065	0.9069
100	0.000180	0.9984	0.000120	0.9343
120	0.000220	0.9909	0.000140	0.9154
140	0.000316	0.9636	0.000150	0.9840
150	0.000412	0.9264	0.000330	0.9676
165	0.001900	0.9980	0.009105	0.9127
180	0.008500	0.9249	0.018780	0.6097

**Table 4:** Arrhenius parameters for reaction between metallic silver and sulfur in mineral insulating oil following first order kinetics.

Temperature, °C	Order of Reaction	A Pre-Exponential Factor	Ea, kJ mol^−1^	R^2^
60		0.3313 0.0845	23.67 21.10	0.9874 0.9194
80				
100	first			
120	second			
140				
150				
Temperature, °C	order of reaction	A pre-exponential factor	Ea, kJ mol^−1^	R^2^
150	first second	2.84 × 10^16^ 2.36 × 10^23^	160.69 216.10	0.9998 0.8919
165				
180				

## Data Availability

The original contributions presented in this study are included in the article. Further inquiries can be directed to the corresponding author.

## References

[1] Holt A.F., Facciotti M., Amaro P., Brown R.C.D., Lewin P.L., Pilgrim J.A. (2013). Silver Corrosion in Transformers. Proceedings of the 2013 Annual Report Conference on Electrical Insulation and Dielectric Phenomena.

[2] Holt A.F., Facciotti M., Amaro P., Brown R.C.D., Lewin P.L., Pilgrim J.A., Wilson G., Jarman P. (2013). An initial study into silver corrosion in transformers following oil reclamation. Proceedings of the 2013 IEEE Electrical Insulation Conference (EIC).

[3] Samarasinghe S., Ma H., Martin D., Saha T. (2019). Investigations of Silver Sulfide Formation on Transformer OLTC Tap Selectors and Its Influence on Oil Properties. IEEE Trans. Dielectr. Electr. Insul..

[4] Martinez R., Fares C.N., Vidal D., Chiarella C. Investigating cause of failure in a 500 kV transmission transformer. Proceedings of the MyTransfo.

[5] Samarasinghe S. Potential Mitigation Strategies to Prevent Silver Sulphide Corrosion in a Transformer OLTC Australian Power Technologies Transmission & Distribution, October–November 2020, Issue 5. https://eecs.uq.edu.au/files/7347/T%26D%20Oct-NOV%202020-our%20articles.pdf.

[6] Scatiggio F., Tumiatti V., Maina R., Pompili M., Bartnikas R. (2007). Corrosive Sulfur in Insulating Oils: Its Detection and Correlated Power Apparatus Failures. IEEE Trans. Power Deliv..

[7] Lewand L., Reed S. Destruction of Dibenzyl Disulfide in Transformer Oil. Proceedings of the 75th Doble Client Conference.

[8] Dukhi V., Bissessur A., Martincigh B. (2016). Formation of Corrosive Sulfur with Dibenzyl Disulfide in Fluid-Filled Transformer. Ind. Eng. Chem. Res..

[9] Ren S., Yang X., Xiaolong C., Lisheng Z., Qinxue Y., Jeanjean R. A Research Summary of Corrosive Sulfur in Mineral Oils. Proceedings of the 2009 IEEE 9th International Conference on the Properties and Applications of Dielectric Materials.

[10] (2009). CIGRE WG A2-32 “Copper Sulphide in Transformer Insulation”, Final Report. https://pdfcoffee.com/378-copper-sulphide-in-transformer-insulation-pdf-free.html.

[11] Toyama S., Tanimura J., Yamada N., Nagao E., Amimoto T. (2009). Highly sensitive detection method of dibenzyl disulfide and the elucidation of the mechanism of copper sulfide generation in insulating oil. IEEE Trans. Dielectr. Electr. Insul..

[12] Amimoto T., Hosokawa N., Nagao E., Tanimura J., Toyama S. (2009). Concentration dependence of corrosive sulfur on copper-sulfide deposition on insulating paper used for power transformer insulation. IEEE Trans. Dielectr. Electr. Insul..

[13] Ren S., Zhong L., Yu Q., Cao X., Li S. (2012). Influence of the atmosphere on the reaction of dibenzyl disulfide with copper inmineral insulation oil. IEEE Trans. Dielectr. Electr. Insul..

[14] Jankovic J., Lukic J., Kolarski D., Veljović D., Radovanović Ž., Dimitrijević S. (2023). Isotherm, Thermodynamic and Kinetic Studies of Elemental Sulfur Removal from Mineral Insulating Oils Using Highly Selective Adsorbent. Materials.

[15] Cigre Working Group A2.40 (2015). Copper Sulphide Long-Term Mitigation and Risk Assessment. CIGRE. https://www.e-cigre.org/publications/detail/625-copper-sulphide-long-term-mitigation-and-risk-assessment.html.

[16] Dahlund M., Johansson H., Lager U., Wilson G. Understanding the presence of corrosive sulphur in previously non-corrosive oils following regeneration. Proceedings of the 77th Annual International Conference of Doble Clients Conference.

[17] Lewand L. (2002). The Role of Corrosive Sulfur in Transformers and Transformer Oil.

[18] Foata M., Lindl K.H., Da Costa M., Lukic J., Jankovic J., Mihajlovic D. Risk Assessment and Mitigation of Corrosive Sulphur Other than DBDS. Proceedings of the CigreBrasil X Workspot.

[19] (2009). Copper Sulphide in Transformer Insulation. CIGRE Technical Brochure 378.

[20] Samarasinghe S., Ekanayake C., Ma H., Saha T.K., Baniya J., Allan D., Russell G. (2022). A Risk Assessment for Utilities to Prevent Transformer OLTC Failures Caused by Silver Sulphide Corrosion. IEEE Trans. Power Deliv..

[21] Foley R.T., Xorrill W., Wisslow S.J. (1950). The mechasism of the reactiox between silver asd sulfur ix mineral oil. J. Phys. Colloid Chem..

[22] Reagor B.T., Sinclair J.D. (1981). Tarnishing of Silver by Sulfur Vapor: Film Characteristics and Humidity Effects. J. Electrochem. Soc..

[23] Huo Y., Fu S.W., Chen Y.L., Lee C.C. (2016). A reaction study of sulfur vapor with silver and silver–indium solid solution as a tarnishing test method. J. Mater. Sci. Mater. Electron..

[24] (2015). Standard Test Method for Corrosive Sulfur in Electrical Insulating Liquids.

[25] (2018). Test Methods for Quantitative Determination of Corrosive Sulfur Compounds in Unused and Used Insulating Liquids—Part 3: Test Method for Quantitative Determination of Elemental Sulfur.

[26] Reinhold H., Möhring H. (1935). Bildungsgeschwindigkeit und elektrische Leitfähigkeit des ß-Schwefelsilbers. Ein Beitrag zur Kenntnis des Anlanfvorganges. Z. Für Phys. Chem..

[27] Wagner C. (1933). Über die Natur des elektrischen Leitvermögens von α-Silbersulfld. Z. Für Phys. Chem..

[28] Lukić J., Mihajlović D., Vasović V., Janković J., Drakić K., Milosavljević S. The Mitigation Techniques on Corrosive Sulphur. Proceedings of the TechCon Asia Pacific.

[29] Lukic J., Jankovic J., Planojevic J., Foata M., Zieglschmid C., Castano V., Briotto A. Silver Sulphide in OLTCs—Root Causes and Proactive Mitigation Strategies. Proceedings of the TechCon, Aus-NZ.

[30] Lukic J., Jankovic J., Planojevic J., Ivancevic V., Milosavljevic S., Foata M., Fleischmann W., Frotscher R. Sulphur Corrosion Mitigation in Power Transformer Life Extension. Proceedings of the 9th CIGRE Workspot.

[31] Sievers C., Noda Y., Qi E.L., Albuquerque R., Scott S. (2016). Phenomena Affecting Catalytic Reactions at Solid-Liquid Interfaces. ACS Catal..

[32] Thoenes D. (1994). The Interaction of Chemical Reactions and Physical Transport Phenomena. Chemical Reactor Development.

[33] Freeman D., Doll J.D. (1983). The influence of diffusion on surface reaction kinetics. J. Chem. Phys..

[34] Bamford C.H. (1985). Diffusion-Controlled Reactions in Solution. Comprehensive Chemical Kinetics.

[35] Baetzold R.C., Somorjai G.A. (1976). Preexponential Factors in Surface Reactions. J. Catal..

[36] Levenspiel O. (1998). Chemical Reaction Engineering.

